# Nasopharyngeal Coil Dislocation of an Embolized Internal Carotid Artery Pseudoaneurysm

**DOI:** 10.1155/2021/4270441

**Published:** 2021-09-11

**Authors:** Nazli Ay, Ingo Todt, Holger Sudhoff

**Affiliations:** Department of Otorhinolaryngology, Head and Neck Surgery, Medical Faculty OWL, Bielefeld University, Campus Klinikum, Bielefeld, Germany

## Abstract

**Objective:**

Severe epistaxis caused by ruptured intracranial pseudoaneurysms can be effectively treated by coil embolization. This is generally an efficient and safe procedure and provides sufficient protection recurrent epistaxis. However, complications such as aneurysm rupture, arterial dissection, bleeding, and emboli can occur. A dislocation of a nasopharyngeal coil is an extremely rare event. *Patient*. We present a case of a 61-year-old patient with a recurrent undifferentiated nasopharyngeal carcinoma (NPC) treated with severe epistaxis. Initially, epistaxis was successfully controlled by a nasal packing. Recurrent bleeding despite packing required a neuroradiological intervention. An intracranially ruptured pseudoaneurysm was detected by magnetic resonance imaging (MRI) and computed tomography (CT), originating from the internal carotid artery at the junction of the petrous part to the cavernous part. Coiling and endovascular plug embolization was performed for the treatment of aneurysm. Ten months later, the patient removed a foreign body out of his left nose. It was dislocated coil material due to radionecrosis. MRI confirmed sufficient embolization of the internal carotid artery.

**Conclusions:**

This case report highlights the possibility of a nasopharyngeal coil dislocation of an embolized internal carotid artery aneurysm emerging as a nasal foreign body.

## 1. Introduction

Intercranial aneurysms leading to severe epistaxis can be treated by microsurgical clipping and/or endovascular embolization. Endovascular embolization has acquired major acceptance due to the progress in endovascular techniques with improved long-term results [1]. Generally, this method produces reliable clinical results providing protection against recurrent epistaxis [2]. The treatment of intracranial aneurysms (IA) aims to prevent rupture and subarachnoid hemorrhage (SAH). IAs are the most common cause of SAH, occurring at an estimated incidence of 6–16 cases per 100,000 people per year [3]. Complications occurring during coil embolization include rupture of aneurysm, arterial dissection, bleeding, and embolic complications. Coil dislocation is another rare complication of coil embolization which appears in 2–6% of these procedures [4]. A dislocated coil can be fixed by retrieval of the coil from the aneurysm or placement of the coil back into the aneurysm. This case highlights a patient with a rare complication after embolization of a ruptured internal carotid artery, a nasopharyngeal coil dislocation a few months after treatment.

## 2. Case Report

**Figure 1:**
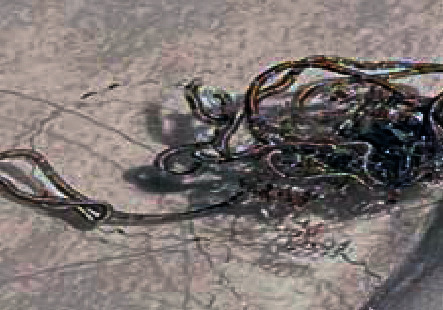
Dislocated removed coil material by the patient from his left nose.

**Figure 2:**
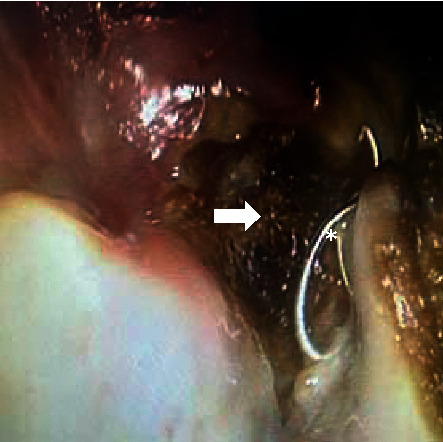
Endoscopic nasopharyngeal view showing the left necrotic lateral area with the dislocated coil (^*∗*^coil, white arrow: necrotic area).

**Figure 3:**
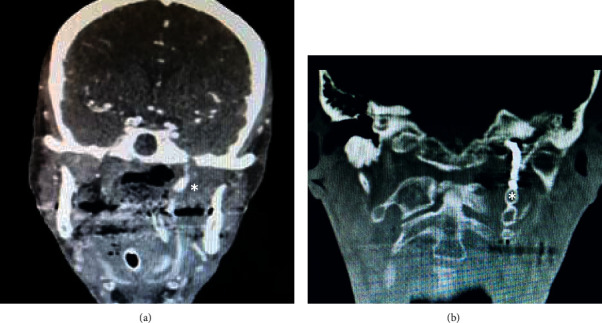
(a-b) Computed tomography after embolization and dislocation of the coil (^*∗*^plug without coil). No extravasation was noted.

**Figure 4:**
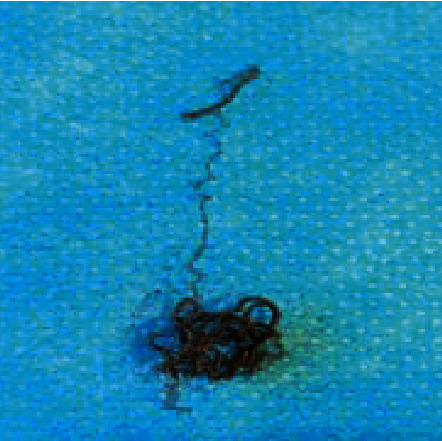
Additional endoscopically removed excess coil material from the exit area in a surgical setting.

A 61-year-old patient with a recurrent radiated undifferentiated nasopharyngeal carcinoma (NPC) was admitted to hospital with severe epistaxis. Initially, epistaxis was successfully controlled by nasal packing. Subsequently, recurrent bleeding despite packing required a neuroradiological intervention. An intracranial ruptured aneurysm was detected by magnetic resonance imaging (MRI) and computed tomography (CT) derived from the internal carotid artery at the junction of the petrous part to the cavernous part. Coiling and endovascular plug embolization was performed for the treatment of the aneurysm by neuroradiology. Ten months later, the patient described fibrous material that he had pulled partly out of his left nose and presented on his handkerchief (Figure 1). It was coil material confirmed by nasal endoscopy (Figure 2). A repeated MRI confirmed persistent sufficient embolization of the internal carotid artery with a vascular plug (Figures 3(a) and 3(b)). No extravasation was noted. The accessible extruded material was divided, leaving the residual material in place in a controlled setting (Figure 4). No further measures were taken. The patient remained stable and revealed no further epistaxis over a 9-month follow-up. Despite of i.v. and long-term oral antibiotics, radionecrosis did not sufficiently heal. Hyperbaric oxygenation was not considered due to the reduced overall condition of the patient. Follow-up endoscopy revealed exposed bone of the cervical spine and granulation tissue at the radiated primary tumor site. Repeated biopsies showed no malignant cells.

## 3. Discussion

Endovascular plug embolization is generally an efficient and safe procedure and provides sufficient protection for recurrent epistaxis [5]. Complications such as aneurysm rupture, arterial dissection, bleeding, and emboli may occur. A dislocation into the nasopharynx of the coil is an extremely rare event. Nasopharyngeal radionecrosis was the etiology of epistaxis and coil dislocation. Our case demonstrated an unexpected complication after coiling and endovascular plug embolization. During the endovascular treatments of intracranial aneurysms, a dislocation of the coil may occur. This is a serious intraprocedural complication. The risk of coil dislocation depends on anatomical and technical factors. In our case, there were necrotic areas around the coiled IA due to high-dose radiotherapy for the NPC. Endovascular plug is a useful adjunct measure, reducing the prevalence of complication. Additionally, it significantly decreases the duration and radiation exposure of the procedure and the number of required coils. Furthermore, coils can potentially generate thrombogenic emboli. Despite embolization, there is a small risk of reperfusion and induction of embolic events. These events can be life-threatening and result in wide brain infarctions. Regular follow-up examinations are necessary.

There is no standard method for dislocated coils retrieval [6]. Various endovascular methods have been reported in a limited number of cases. Some selected cases may require a recovery of the dislocated coil. However, it can be challenging to harvest the protruding end of the coil. Manipulation can potentially induce vascular spasms and pose further complications. Notably, radionecrosis and infection can be directly related to extravascular coil dislocation.

In our presented case, there was no necessity to remove the residual coil as the vascular plug still kept the internal carotid artery closed and not perfused.

## 4. Conclusions

This case report highlights the possibility of a nasopharyngeal coil dislocation of embolized internal carotid artery aneurysm due to radionecrosis. The coil presented as a nasal foreign body. Dislocated coils should only be removed in a controlled clinical setting.

## Data Availability

The data used to support the findings of this study are included within the article.
